# Environment-friendly, high-performance cellulose nanofiber-vanillin epoxy nanocomposite with excellent mechanical, thermal insulation and UV shielding properties

**DOI:** 10.1016/j.heliyon.2024.e25272

**Published:** 2024-01-24

**Authors:** Bijender Kumar, Samia Adil, Duc Hoa Pham, Jaehwan Kim

**Affiliations:** Creative Research Center for Nanocellulose Future Composites, Department of Mechanical Engineering, Inha University, 100, Inha-ro, Michuhol-gu, Incheon, 22212, South Korea

**Keywords:** Bio-based epoxy, Cellulose nanofiber, Nanocomposites, Mechanical properties, UV shielding, Thermal insulation

## Abstract

With the increased demand for biobased epoxy thermosets as an alternative to petroleum-based materials in various fields, developing environment-friendly and high-performance natural fiber-biobased epoxy nanocomposites is crucial for industrial applications. Herein, an environment-friendly nanocomposite is reported by introducing cellulose nanofiber (CNF) in situ interaction with lignin-derived vanillin epoxy (VE) monomer and 4, 4´-diaminodiphenyl methane (DDM) hardener that serves as a multifunctional platform. The CNF-VE nanocomposite is fabricated by simply dispersing the CNF suspension to the VE and DDM hardener solution through the in-situ reaction, and its mechanical properties and thermal insulation behavior, wettability, chemical resistance, and optical properties are evaluated with the CNF weight percent variation. The well-dispersed CNF-VE nanocomposite exhibited high tensile strength (∼127.78 ± 3.99 MPa) and strain-at-break (∼16.49 ± 0.61 %), haziness (∼50 %) and UV-shielding properties. The in situ loading of CNF forms covalent crosslinking with the VE and favors improving the mechanical properties along with the homogeneous dispersion of CNF. The CNF-VE nanocomposite also shows lower thermal conductivity (0.26 Wm^−1^K^−1^) than glass. The environment-friendly and high-performance nanocomposite provides multiple platforms and can be used for building materials.

## 1 Introduction

Cellulose is one of the plant's main components, which provides higher mechanical properties to the wood where lignin and cellulose bind with the hemicellulose linker [1]. Recently, cellulose nanocrystal (CNC) and cellulose nanofiber (CNF), so-called nanocellulose, derived from biobased materials have attracted significant interest as building blocks for developing high-performance green materials owing to their abundance, renewability, lightweight, high mechanical properties, and biodegradability [[2], [3], [4], [5], [6]]. Due to its high aspect ratio and flexibility, CNF has recently gained significant attention due to its potential to replace synthetic fibers in various applications [7]. CNF is a promising candidate in green composite materials [8].

A biomass-derived epoxy has multifold advantages, such as being environmentally friendly, chemical resistant, and having superior mechanical and thermal properties [[9], [10], [11]]. In recent decades, various biobased epoxy thermosets and their composites from natural resources have been explored to reduce greenhouse gas emissions [[12], [13], [14], [15]]. Owing to the high availability of lignin, being a wood-based material, lignin-derived epoxy resins attract the most attention as a constituent in green composite materials [[16], [17], [18]]. Vanillin, a non-toxic monoaromatic organic compound, can be prepared from lignin at the industrial scale, and synthesizing epoxy resins from it is convenient instead of using lignin directly [9]. Several studies have addressed the synthesis of vanillin-based epoxy thermosets from a renewable source [11,19,20]. Unfortunately, most biobased thermosets exhibit brittleness, low toughness, and limited strength, significantly lower than commercial thermosets [14,21].

CNF and epoxy nanocomposites have attracted the research community significantly due to their extensive use in broad applications, including adhesives, coatings, sporting goods, UV shielding, and electronics [3,22]. The high concentration loading of CNC in epoxy improved mechanical properties and water-resistant materials. The CNC–OH was crosslinked with epoxy through etherification, resulting in the nanocomposite's high mechanical properties [23]. Saba and his co-workers investigated the effect of CNF loading percentage in epoxy. They achieved the highest mechanical properties at 0.75 % CNF [8]. Recently, Park et al. fabricated the CNF/epoxy nanocomposites by impregnating CNF with epoxy resins [24]. The nanocomposites showed transmittance higher than 90 %, and the haziness was less than 5 %. In contrast, the strength and stiffness of the nanocomposite were not significantly improved. However, most CNF or CNC-epoxy nanocomposites have been made using commercial petroleum-based epoxies for numerous applications [2,8,22,23,25,26].

A greater emphasis is given in the literature on high-performance and environment-friendly CNF-biobased epoxy nanocomposites because using biobased CNF and epoxy materials can mitigate greenhouse gas emissions. Due to severe climate change, we should use environment-friendly materials that satisfy net-zero emissions. However, limited reports have been made on incorporating CNF into biobased epoxy to fabricate environment-friendly nanocomposites for advanced applications. These nanocomposites are usually composed of natural fibers and biobased epoxy resins. The advantages of these nanocomposites include satisfying net-zero emissions, high biodegradability, no/less toxicity, and excellent environment-friendliness. CNF incorporation into biobased epoxy can lead to lightweight, transparent, high-performance nanocomposites that can be desirable for automobiles, engineering structures, and replacing plastics or glass [24,27]. Subbotina and his co-workers recently designed environment-friendly nanocomposites by impregnating diluted biomass-derived epoxy solution into colloidal CNF suspension [28]. They found that the biocomposite *T*_*g*_ increased compared to the epoxy matrix due to the CNF's –OH groups covalently linked to the epoxy ring.

Based on the abovementioned considerations and the scarcity of nanocomposites' flexibility, a lignin-derived vanillin epoxy (VE) thermoset is selected to fabricate the environment-friendly, strong and tough CNF-VE nanocomposites. The choice of lignin-derived VE is based on its resource from the second most abundant lignin on the earth. Besides, the VE thermoset has some interesting properties compared to CNF - higher thermal stability, hydrophobicity, chemical resistance, and UV absorbing performance [19,21,29]. To the best of our knowledge, there is no research on the CNF loading into the lignin-derived VE to obtain the environment-friendly nanocomposites, which endow hydrophobicity, UV shielding, high-mechanical properties, and heat management properties for high-value structural applications.

Herein, lignin-derived VE was prepared by the reaction of vanillin and epichlorohydrin in the presence of NaOH and phase transfer catalyst, tetrabutylammonium bromide (TBAB). Further, a solvent-exchange methodology was adopted to incorporate CNF into the lignin-derived VE and 4, 4´-diaminodiphenyl methane (DDM) hardener resin through the in situ reaction to develop novel high-performance and environment-friendly CNF-VE nanocomposites. The high aspect ratio, ductile nature, high strength, and modulus of CNF lead to fabricating high-performance, environment-friendly nanocomposites. Different % of CNFs are incorporated into the resin to enhance the strength and modulus of the nanocomposites. We systematically studied the effect of CNF loading on the tensile strength, fracture morphology, thermal stability, UV shielding, and wettability. The interfacial interaction of CNF with VE-DDM thermoset-containing imine bond and dispersion into a matrix was also studied. An imine bond network containing epoxy thermosets can undergo the hydrolysis reaction in an acidic medium due to the imine bond's reversibility. Thus, the degradation behavior of CNF-VE nanocomposites was studied in organic solvents and acidic conditions.

## 2 Experimental

### 2.1 Materials

Cellulose nanofibers (CNFs, 2.0 wt%) were procured from Moorim Paper MFG Co., Ltd., South Korea. CNF suspension of 1 mmol/g with an average width of less than 100 nm and length of up to several micrometers. Fig. S1 (Supporting *Information*) represents the CNFs' TEM image and the length distribution. The width and length were measured from the TEM image. Vanillin (98 %), epichlorohydrin (ECH, 99 %), tetrabutylammonium bromide (TBAB, ≥99 %), 4, 4´-diaminodiphenyl methane (DDM, ≥97 %) were received from Sigma Aldrich. Anhydrous sodium sulfate (99 %) was procured from Yakuri Pure Chemicals Co., Ltd., Japan. Dichloromethane (DCM, >99.9 %) was obtained from Samchun, South Korea. Acetone (99.5 %), sodium hydroxide (97 %), and ethanol (99.5 %) were obtained from Daejung Chemical and Metals Co., Ltd., South Korea.

### 2.2 Preparation of vanillin epoxy thermoset

**Fig. 1:**
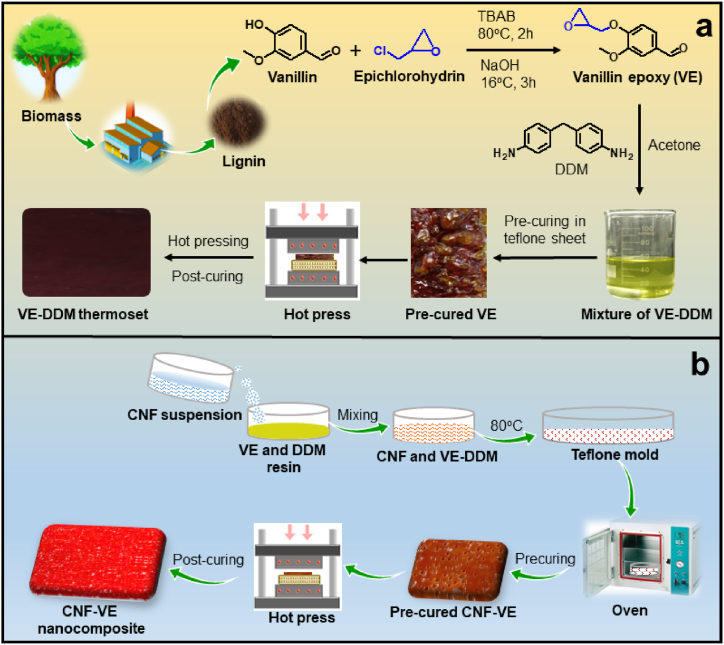
Schematic representation of the preparation methodology of (a) VE and VE-DDM thermoset and (b) CNF-VE nanocomposite.

VE resin and cured thermoset were synthesized according to the following procedure [30,31]: first, vanillin (5.0 g) was dissolved in ECH (12.0 g) into the round bottom flask, and then TBAB (5 wt% of vanillin) was mixed and heated at 80 °C for 2 h, as shown in Fig. 1a. Afterward, 50 wt% of NaOH aqueous solution (3.3 g) was added dropwise into the reaction mixture within 5 min at 16 °C and continued the reaction for 3 h. The obtained mixture was filtered, thoroughly washed with water to remove impurities, and dried at 80 °C for 2 h. The yield of VE was 80.4 %.

Next, to obtain the VE-DDM thermoset, VE (5.0 g) and DDM (3.56 g) were dissolved in acetone. After complete dissolution, the acetone was evaporated at 80 °C in the oven. The pre-curing of the epoxy mixture was carried out at 100 °C and 140 °C for 1 h each. Further, in a hot-press machine, biobased epoxy was hot-pressed at 180 °C for 10 min (2 min for degassing and 8 min for pressing) and post-cured at 150 °C and 170 °C for 1 h each in an oven (Fig. 1a).

### 2.3 Preparation of solvent-exchange CNF

Solvent-exchange CNF was prepared by centrifuging a 2.0 wt% suspension of aqueous CNF. The 2.0 wt% CNF (100.0 g) was mixed in 500 mL acetone, and the mixture was centrifuged at 10,000 rpm for 10 min to exchange the water to obtain the agglomerated CNF. After separating the supernatant, the acetone was added to the agglomerated CNF and centrifuged to exchange the remaining water. Finally, the agglomerated CNF was stored in the freezer. The agglomerated CNF is shown in Fig. S2.

### 2.4 In situ fabrication of CNF-VE nanocomposite

CNF-VE nanocomposites were fabricated by simply dispersing the CNF suspension to the VE and DDM hardener solution through the in-situ reaction, as shown in Fig. 1b. Firstly, VE and DDM hardeners were dissolved in acetone, in which epoxy and amine were in a 4:3 mol ratio. Different wt% of the agglomerated CNF (0.5, 0.75, 1.0, and 1.5 wt% CNFs) was dispersed in acetone and homogenized at 8000 rpm for 10 min to obtain the uniform dispersion CNF suspensions. Subsequently, the CNF suspensions were mixed with the VE and DDM solution. Next, the mixture was placed in an oven at 80 °C to remove the solvent, followed by pre-curing at 100 °C and 140 °C for 1 h each. Further, pre-cured CNF-VE was kept in a hot plate of the hot-press machine at 180 °C for 2–3 min to remove the air bubbles. After that, it was hot-pressed for 7 min. Then, the sample was post-cured again at 150 °C and 170 °C for 1 h each in an oven. Depending on the wt% of CNF, the CNF-VE was named 0.5CNF-VE, 0.75CNF-VE, 1.0CNF-VE, and 1.5CNF-VE nanocomposites.

### 2.5 Characterization

The VE resin and cured VE-DDM thermoset functional groups and their CNF interactions were investigated using FTIR spectroscopy (Cary 630, Agilent Technologies, USA). ^1^H and ^13^C NMR spectra were taken to ensure the chemical structure of synthesized VE resin using an NMR spectrometer (Bruker Avance III 400 MHz, USA), and DMSO was used as a solvent. The thermal properties of all the samples were examined using a thermogravimetric analyzer (TGA, TG 209F3, Netzsch, Selb, Germany) in a nitrogen atmosphere. The glass transition temperature (*T*_*g*_) of the VE thermoset and CNF-VE nanocomposites were analyzed by differential scanning calorimetry (DSC, 200 F3 Maia, Netzsch, Selb, Germany). The stress-strain curves of the VE-DDM thermoset and CNF-VE nanocomposites were recorded by a tensile test machine (TEST ONE, TO-100-IC, South Korea) at room temperature. All samples were tested with the dimensions (length 50 mm, width 2 mm, thickness 0.45 mm). The mechanical properties of the wet CNF-VE nanocomposite were examined using the same tensile test machine. The CNF-VE nanocomposites' fracture surfaces were examined using a scanning electron microscope (SEM, S-4000, Hitachi, Japan). The prepared resin's water contact angle (WCA) was measured using a WCA measurement system (GSA, Surfacetech Co., South Korea). The same tensile test machine was used for measuring the interfacial shear strength of VE-DDM resin with CNF film through the shear lap joint (SLJ) test (length 40 mm, width 10 mm). Fig. S3 represents the SLJ test procedure. The water absorption of the CNF-VE nanocomposites (dimensions: 50 mm × 2 mm × 0.45 mm) was measured for short-term immersion (7 days) in tap water at RT. The water absorption of samples was measured after wiping out water. The thermal insulation performance, namely, thermal conductivity (λ) and thermal diffusivity (α) of VE-DDM thermoset and CNF-VE nanocomposites, were analyzed using a thermal constants analyzer (TPS 2500S, Hot Disk AB, Sweden) at room temperature with an output power of 13 mW for 5 s according to the ISO 22007–2:2015 standard, followed by the transient plane source (TPS) method [32]. A nickel spiral sensor (Kapton 7577, radius 2.001 mm) and a resistant thermometer were placed between two identical rectangular samples (thickness 10 mm).

### 2.6 Statistical analysis

The statistical analysis was performed using the SPSS statistical analysis program (SPSS Inc., Chicago, IL, USA). The one-way analysis of variance (ANOVA) was performed on SPSS to determine the significance of each mean property by post hoc Duncan's multiple range test (p < 0.05).

## 3 Results and discussions

### 3.1 Characterization of VE and CNF-VE nanocomposites

Biobased VE was prepared by the reaction of vanillin and epichlorohydrin, as shown in Fig. 1a. NMR characterization was carried out to confirm the structure of VE before preparing the CNF-VE nanocomposites. In the NMR spectrum of the VE (Fig. S4), the signals for H1, H2, and H3 confirm the presence of an epoxy ring. The H4 signal is attributed to –OCH_3_, H8 corresponds to –CH=O, and H5, H6, and H7 proton signals belong to the aromatic skeleton. As shown in ^13^C NMR (Fig. S5), the chemical shifts at 44.29 ppm, 49.94 ppm, and 70.40 ppm also confirm the epoxy ring and the number of carbon signals are the same as the carbon of the prepared VE.

**Fig. 2:**
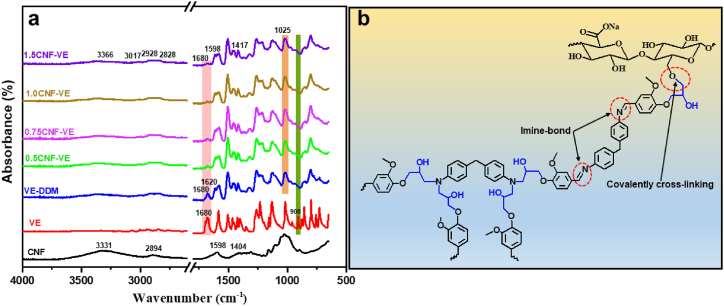
FTIR spectra of the (a) CNF, prepared VE, VE-DDM, CNF-loaded VE-DDM thermoset and (b) representative chemically interaction diagram of CNF-VE nanocomposites.

Fig. 2a shows the FTIR spectra of CNF-VE nanocomposites, the pristine CNF, VE resin, and VE-DDM thermoset. CNF's characteristic absorption peaks at 3331 cm^−1^, 1598 cm^−1^, and 1025 cm^−1^ correspond to the stretching vibration of –OH, carboxylate group, and ether (–CH_2_-*O*–CH_2_–) group, respectively [33]. The absorption band at 908 cm^−1^ corresponds to the epoxy ring, and the band at 1680 cm^−1^ belongs to the formyl group in the FTIR of VE resin. However, the absorption bands of the epoxy ring in the cured VE-DDM thermoset were consumed entirely after curing with the hardener. A new absorption band for the C=N bond at 1620 cm^−1^ is due to the reaction between the formyl (-CH=O) group of VE and the –NH_2_ group of hardener [30,31]. Another noticeable change is the decrease in the band intensity of the formyl group after the curing, suggesting the successful completion of the curing between the epoxy and the hardener. With increasing the CNF content to 0.75 wt% in the mixture of VE and hardener, the peak intensity of the -C-*O*-C group at 1025 cm^−1^ increased gradually compared to the VE-DDM thermoset after curing the CNF-VE nanocomposites. It is due to the etherification crosslinking of CNF with an epoxy ring (Fig. 2b), and the crosslinking of the VE and DDM hardener is shown in Fig. S6 [28]. However, with the loading CNF of more than 0.75 wt%, the peak intensity slightly decreased due to the intramolecular hydrogen bonding of CNFs′ hydroxyl groups, resulting in a lower etherification reaction. Moreover, an epoxy ring's absorption bands at 908 cm^−1^ disappeared entirely after curing the CNF-VE nanocomposites. These changes confirm the successful curing of CNF-VE nanocomposites.

### 3.2 Mechanical properties of VE-DDM and CNF-VE nanocomposites

**Fig. 3:**
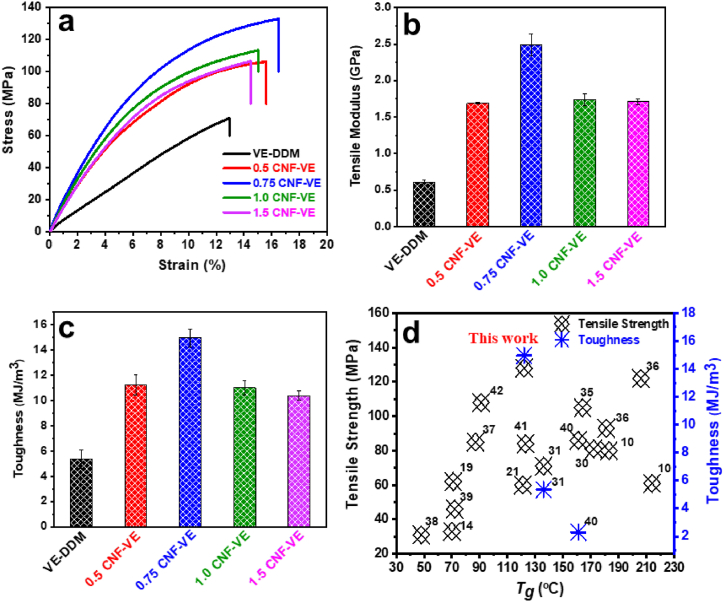
Stress-strain curve of the (a) VE-DDM thermoset and CNF-VE nanocomposites, (b) Tensile modulus, (c) Toughness, and (d) This work comparison with previously reported literature on the vanillin-based epoxy thermoset and natural fiber-reinforced composites.

Fig. 3 shows the mechanical properties of the VE-DDM thermoset and CNF-VE nanocomposites. The corresponding tensile strength, Young's modulus, toughness, and elongation at break values are presented in Table 1. Fig. 3a illustrates the stress-strain curves of the nanocomposites with various CNF loadings. The nanocomposite containing 0.75 wt% CNF displayed the highest tensile strength (127.78 ± 3.99 MPa), Young's modulus (2.49 ± 0.15 GPa) (Fig. 3b), and toughness amongst all the nanocomposites. At lower loading of CNF, the nanocomposite showed excellent CNF dispersion and good compatibility with the epoxy matrix. The highest mechanical properties for 0.75CNF-VE nanocomposite can be attributed to the robust covalent-crosslinking interaction of CNF's hydroxyl groups with the epoxy ring (Fig. 2b) and the homogeneous in-situ dispersion of CNF into the epoxy resin along with the good interfacial adhesion strength [34]. In our previous study, the VE-DDM resin showed good interfacial adhesion around 13.93 ± 0.26 MPa with CNF film (Fig. S7) because of its excellent compatibility and crosslinking [31]. The underlying mechanisms for the increased mechanical properties are etherification and crosslinked network structure. With the loading of 0.75CNF (Fig. 3c), the toughness of the nanocomposites is improved significantly due to the good interfacial bonding induced plastic behavior of CNF with epoxy. Higher loading of CNF promoted the reduction in the toughness of the composites due to the fibers' agglomeration and poor crosslinking of CNF with epoxy. The mechanical properties of the nanocomposites are higher than many biobased vanillin epoxy thermosets [10,14,19,21,30,31,[35], [36], [37], [38], [39], [40]] and natural fibers-reinforced composites [41,42], as delineated in Fig. 3d. When the CNF concentrations increased, the mechanical properties dropped gradually due to the fibers' agglomeration and poor fiber-matrix crosslinking [14,43]. At the 0.75 wt% CNF loading, 0.75CNF-VE nanocomposite showed a higher tensile strength of 127.78 ± 3.99 MPa and Young's modulus around ∼2.49 ± 0.15 GPa.

**Table 1:** Mechanical properties of VE-DDM and CNF-VE nanocomposites.

Samples^a^	Stress (MPa)	Strain (%)	Tensile modulus (GPa)	Toughness (MJ/m^3^)
VE-DDM	70.13 ± 2.95^a)^	12.33 ± 1.8^a)^	0.60 ± 0.05^a)^	5.35 ± 0.75^a)^
0.5CNF-VE	103.35 ± 3.97^b)^	15.6 ± 0.84^b)^	1.69 ± 0.011^b)^	11.24 ± 0.81^b)^
0.75CNF-VE	127.78 ± 3.99^d)^	16.49 ± 0.61^b)^	2.49 ± 0.15^c)^	14.96 ± 0.71^c)^
1.0CNF-VE	114.85 ± 1.97^c)^	14.9 ± 0.72^b)^	1.74 ± 0.08^b)^	11.04 ± 0.57^b)^
1.5CNF-VE	106.52 ± 4.28^b)^	14.58 ± 0.37^b)^	1.71 ± 0.04^b)^	10.39 ± 0.34^b)^

a Same superscript letter data within the same column are not significantly (p > 0.05) different from Duncan's multiple range tests.

### 3.3 Fracture morphology of VE-DDM and CNF-VE nanocomposites

**Fig. 4:**
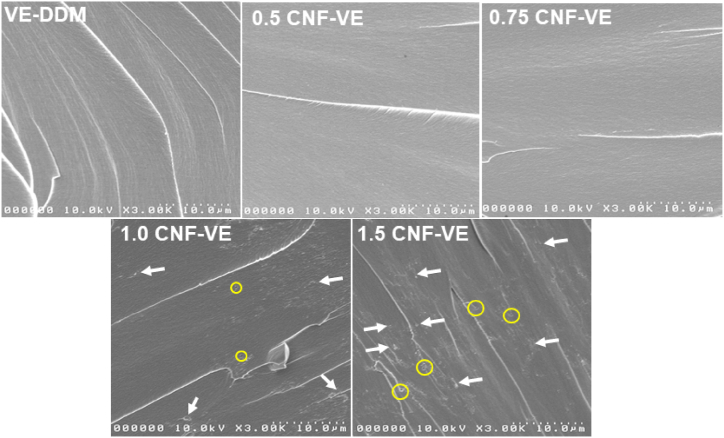
SEM fracture surface morphology of VE-DDM thermoset and CNF-VE nanocomposites (white arrows show CNF agglomerations and yellow circles indicate micro-voids). (For interpretation of the references to colour in this figure legend, the reader is referred to the Web version of this article.)

SEM images of the fractured samples of the VE-DDM thermoset and CNF-VE nanocomposite are illustrated in Fig. 4. The VE-DDM thermoset shows a smooth surface morphology with a glassy exterior and wavy stream crack, indicating its weak resistance to cracking. Nanocomposites up to 0.75 wt% CNF loading exhibit a flat fractured surface with a less glassy exterior than the VE-DDM sample. Further, CNF-VE nanocomposites with 1 and 1.5 wt% CNF loadings display a very different morphology, where CNF agglomerations with a lumpy surface and glassy exterior, voids, and rough surface can be seen. These results further prove that the 0.75 wt% CNF loading nanocomposite possesses the best compatibility with VE-DDM resin. In the case of higher wt% CNF loading, the hydroxyl groups of CNFs make the intramolecular hydrogen bond, resulting in more accumulation of CNFs in the epoxy matrix at certain places. Thus, 0.75CNF-VE nanocomposite exhibits the highest tensile strength, toughness, and Young's Modulus. Similar SEM observations were reported at the optimum wt% of fibers for the fracture surface of vanillin alcohol epoxy/lignin-containing cellulose nanofibrils nanocomposites and CNF-DGEBA nanocomposites [8,14]. In the nanocomposites of higher CNF loading, i.e., 1, 1.5 wt%, the wavy surfaces with more cracks and white dots indicate the agglomerations of the CNFs (white arrows in Fig. 4) and formation of micro-voids (yellow circles in Fig. 4) due to inhomogeneous mixing and poor filler-matrix compatibility. Thus, the 1.5CNF-VE nanocomposite shows the lowest mechanical properties.

### 3.4 Thermal properties

**Fig. 5:**
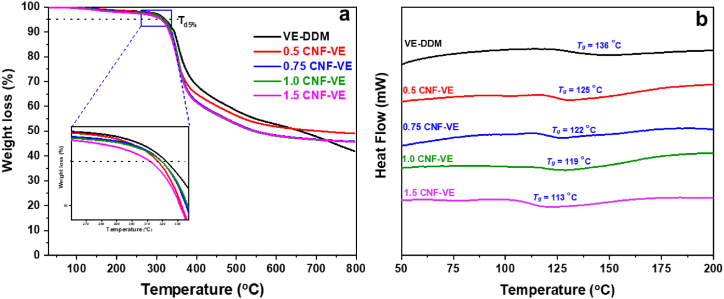
Thermal stability of VE-DDM and CNF-VE nanocomposites (a) TGA and (b) DSC analysis.

TGA and DSC were performed to determine the prepared nanocomposites' thermal properties. As can be seen, all thermosets exhibited one-step degradation (Fig. S8). The primary onset thermal degradation of CNF was at around 203 °C, with a weight loss of 8.24 %, as shown in Fig. S9. The VE-DDM thermoset showed onset thermal degradation at 282 °C with a weight loss of around 2.09 %. In contrast, the degradation weight loss % of CNF-VE nanocomposites increased with the increase of CNF loading, as shown in Fig. 5a and Table 2. It might be due to the decomposition of glycosyl groups of CNF [26]. Nevertheless, the onset thermal degradation of CNF-VE nanocomposites was obtained almost similar to the VE-DDM thermoset, except for the 1.5CNF-VE nanocomposite (⁓276 °C). Besides, the nanocomposites did not show much deviation in the decomposition temperature at T_d5 %_. The covalent crosslinking between the –OH groups of CNF and epoxy ring endows the nanocomposites with almost the same thermal stability. Compared to the epoxy matrix (VE-DDM thermoset), the higher char residue for the nanocomposites is due to the higher degree of crosslinking of the aromatic Schiff base at higher temperatures region, resulting in the formation of the nitrogen-containing ring, and it can lead to making a compact char layer [30,44].

**Table 2:** Thermal and wettability properties of VE-DDM and CNF-VE nanocomposites.

Samples^a^	T_d5 %_ (^o^C)	Weight loss % at onset temperature	*T*_*g*_ (^o^C)	Water contact angle (^o^)	Water absorption (%)
VE-DDM	322	2.09	136	90.41 ± 1.1^a)^	0.96 ± 0.018^a)^
0.5CNF-VE	319	2.36	125	91.53 ± 1.5^a)^	1.23 ± 0.021^b)^
0.75CNF-VE	316	2.64	122	91.79 ± 0.7^a)^	1.26 ± 0.025^b)^
1.0CNF-VE	318	2.81	119	92.05 ± 1.4^a)^	1.39 ± 0.027^c)^
1.5CNF-VE	312	3.05	113	92.18 ± 1.7^a)^	1.46 ± 0.048^d)^

a Same superscript letter data within the same column are not significantly (p > 0.05) different from Duncan's multiple range tests.

Fig. 5b delineates the glass transition temperature (*T*_*g*_) of the VE-DDM thermoset and CNF-VE nanocomposites. The fluctuation in a straight line to down on a heat flow-temperature curve is assumed to be the corresponding *T*_*g*_ of that material. As shown in Fig. 5b, the *T*_*g*_ of the CNF-VE nanocomposites shifted towards the lower temperature with the CNF loading (Table 2). Generally, the covalent crosslinking of fibers with epoxy would result in higher *T*_*g*_ [28,45]. However, the intermolecular hydrogen bonding crosslinking and –OCH_3_ group is expected to promote molecular mobility to the CNF-VE nanocomposites, decreasing *T*_*g*_.

### 3.5 Wettability

**Fig. 6:**
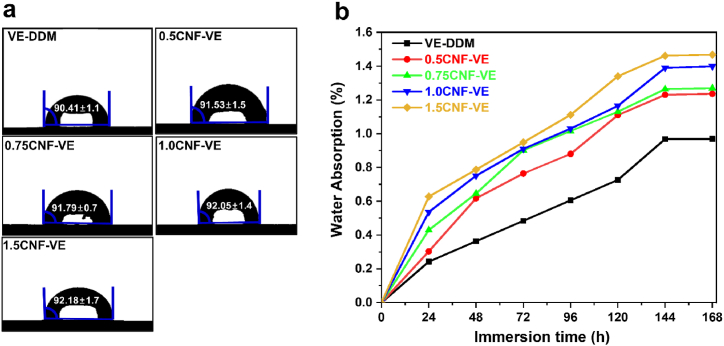
VE-DDM thermoset and CNF-VE nanocomposites: (a) Water contact angles and (b) Water absorption.

Wettability is one of the important parameters in many structural applications, deteriorating materials' performance [46]. We incorporated the CNFs into the hydrophobic VE-DDM matrix using in situ methodology to fabricate high-performance nanocomposites. Interestingly, all CNF-VE nanocomposites exhibited a similar WCA to the VE-DDM thermoset, as shown in Fig. 6a. It indicates that the hydrophobicity of all CNF-VE nanocomposites is similar after incorporating the CNFs into an epoxy matrix (Table 2). Although CNF is a highly hydrophilic material, and its incorporation into the matrix is expected to lower the hydrophilicity of the nanocomposites further, no such effect was observed for any of the nanocomposites because most –OH groups of the CNFs consumed during the crosslinking with epoxy.

Further, the water absorption behavior of VE-DDM and CNF-VE nanocomposites was studied by immersing the samples in tap water for 7 days at room temperature. Initially, the water absorption percentage of VE-DDM and CNF-VE nanocomposites gradually increased with the immersion time (Fig. 6b). However, after 6 days, the water absorption of all thermosets reached the equilibrium point. Compared to the pristine resin, the higher water absorption for CNF-VE nanocomposites could be due to micropores and voids providing access (through the capillary phenomenon) to the water molecules to go inside and be absorbed (Table 2).

Further, we evaluated the mechanical properties to check the nanocomposites' durability after immersion of CNF-VE nanocomposites in tap water. The stress-strain curve is shown in Fig. S10, and the mechanical properties of the nanocomposites are elaborated in Table S1. The tensile strength, modulus, strain, and toughness of the CNF-VE nanocomposites are decreased due to the slight water absorption. The tensile strength of the optimized 0.75CNF-VE nanocomposite dropped from 127.78 ± 3.99 MPa to 119.36 ± 3.58 MPa), and the tensile modulus from 2.49 ± 0.15 GPa to 2.09 ± 0.23 GPa.

### 3.6 Solvent resistance

**Fig. 7:**
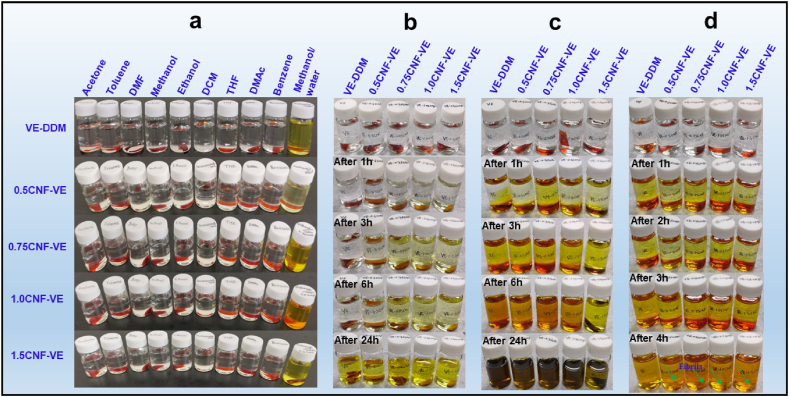
Solvents resistance behavior of VE-DDM and CNF-VE nanocomposites in (a) different solvents and the mixture of methanol/0.1 M HCl aqueous solution at RT, (b) methanol/0.1 M HCl at 60 °C, (c) methanol/0.5 M HCl at 60 °C, and (d) methanol/0.75 M HCl aqueous solution at 60 °C.

Vanillin-derived epoxy thermosets containing a Schiff base (-C=N) polymeric crosslinked network structure tend to have degradation properties in an acidic medium. It can show the reversible covalent reactivity in organic solvents via transamination and imine metathesis [20,21,47,48]. Chemical resistance is an important feature for many applications, and it can influence efficiency and life cycle. Thus, we performed the chemical resistance test of CNF-VE nanocomposites in acidic conditions and organic solvents at room temperature and 60 °C. Fig. 7a demonstrates the digital photographs of the VE-DDM and CNF-VE nanocomposites. All nanocomposites were kept in different organic solvents: acetone, toluene, DMF, methanol, ethanol, THF, DMAc, benzene, and methanol/0.1 M HCl solution (ratio 8:2) at room temperature for 24 h. As presented in Fig. 7, all CNF-VE nanocomposites exhibited good resistance to organic solvents; however, some fractions of samples were dissolved in the mixture of methanol and 0.1 M HCl solution. Note that a previous study showed the entire degradation of the cured VE with 4, 4′- methylenebiscyclohexanamine (PACM) in the methanol/0.1 M HCl solution mixture within 233 min [30]. In contrast, the VE-DDM and CNF-VE nanocomposites were not fully degraded in the methanol and 0.1 M HCl solution (8:2 ratio) until 24 h. It might be due to the more robustly crosslinked aromatic structure of the VE-DDM than the VE-PACM thermoset. The intermolecular hydrogen bonding and etherification crosslinking between CNF–OH and epoxy ring typically makes the nanocomposites solvent-resistant. Thus, despite the imine bond-containing network structure, VE-DDM and CNF-VE nanocomposites showed solvent resistance stability in acidic conditions at ambient temperature. When tested the degradability behavior of samples in methanol/HCl solution (8:2 ratio, with different acidic conditions 0.1 M, 0.5 M, and 0.75 M HCl) mixture at 60 °C, the VE-DDM and CNF-VE nanocomposite displayed a little initial degradability but were not completely degraded (Fig. 7b–c). Interestingly, when increasing acidity to 0.75 M HCl, the degradation rate of VE-DDM and CNF-VE nanocomposite is much higher due to the sufficient acidity/water for hydrolysis reaction [30,49]. Thus, the VE-DDM and CNF-VE nanocomposite thermoset was degraded entirely in methanol/0.75 M HCl solution within 4 h, as shown in Fig. 7d. The CNFs did not degrade in the 0.75 M HCl aqueous solution and remained in the form of agglomerated in the sample vials. This degradability trend of thermoset resin can explore the reusability of all-green natural fiber-polymer composites to recover natural fibers, although the acidic degradation of the nanocomposites can be responsible for the lower performance of recovered natural fibers. Thus, it is a big challenge to intact recovery of natural fibers without degrading the mechanical performance of all-green natural fiber-polymer composites. Based on the results of solvent resistance, the CNF-VE nanocomposite can be used as a paint for building materials applications.

### 3.7 Transmittance, UV shielding, and haze properties

**Fig. 8:**
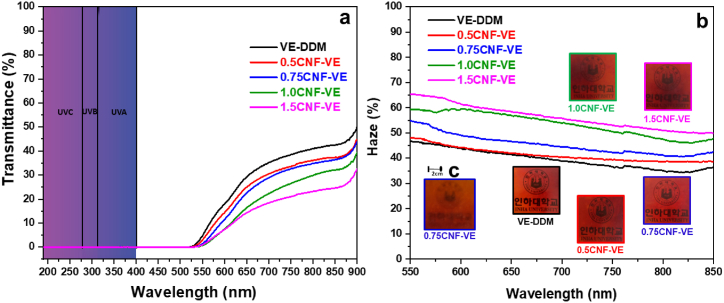
(a) Transmittance and UV-shielding performance. (b) Haze value and the digital images of VE-DDM thermoset and CNF-VE nanocomposites. (c) Inset image of 0.75CNF-VE nanocomposite when it was fixed 2 cm far from the underlying logo.

The optical properties of the VE-DDM and CNF-VE nanocomposites were carried out by UV–Vis spectroscopy, as shown in Fig. 8. The resistance to long-term UV- radiation is a crucial material property for solar panels and outdoor applications [50,51]. It is known that long-term UV exposure can reduce the service life of materials [19]. Thus, developing UV-resistant materials is challenging because of haziness. As presented in Fig. 8a, the VE-DDM exhibited transmittance around ∼29.3 % at 650 nm. With the increase of CNF loading in the matrix, the transmittance of CNF-VE nanocomposites gradually decreased. It might be due to the degree of agglomeration of CNFs with the increasing CNF loading, resulting in scattering and diffraction of UV light. 0.75CNF-VE nanocomposite showed transmittance around ∼22.9 % at 650 nm. Besides the lower transparency, 0.75CNF-VE nanocomposite exhibited a considerably translucent behavior, as shown in the insets of Fig. 8b. Interestingly, when it was 2 cm far from the underlying logo image, the logo was visually obscured (Fig. 8c). The 0.75CNF-VE nanocomposite can protect privacy in high-performance building materials such as windows. Additionally, it showed haziness at around 55 % at 550 nm, although for VE-DDM, it was around 46.5 %. As demonstrated in Fig. 8b, the haze value increased with increasing the CNF loading: it increased to 41.5 % with the 1.5 wt% CNF loading due to the CNF agglomeration in the nanocomposite, resulting in increased scattering and reflecting the UV light. Moreover, CNF-VE nanocomposites and the VE-DDM demonstrate excellent UV-shielding performance in UVA, UVB, and UVC regions, as shown in Fig. 8a. The imine bonds (C=N) of the VE-DDM and CNF-VE nanocomposites′ network structure effectively absorb UV light in UVA, UVB, and UVC regions [19,52]. All the unique features, such as haziness, low transmittance, and UV shielding, suggest that CNF-VE nanocomposites can be used for building materials.

### 3.8 Thermal insulation properties

**Fig. 9:**
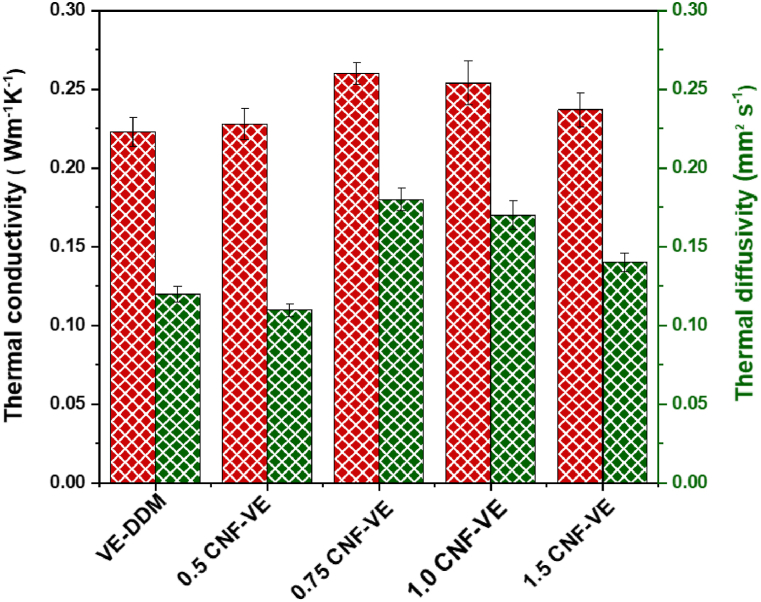
Thermal insulation behavior of VE-DDM thermoset and CNF-VE nanocomposites.

Thermal conductivity is the materials' most significant property determining their applications [53,54]. Good insulating materials can keep the building warm and help store food items longer [55]. Thus, a material with good insulating properties has a significant market value and demand. To examine the heat transfer performance, the thermal conductivity (λ) of the VE-DDM and CNF-VE nanocomposites was measured using the thermal sensor along the in-plane direction at 25 °C. Thermal conductivity (λ) determines the possibility of heat transfer, whereas thermal diffusivity (α) measures the heat transfer rate through that material [56]. As demonstrated in Fig. 9, the VE-DDM thermoset shows a thermal conductivity of 0.223 Wm^−1^K^−1^ and a thermal diffusivity of 0.12 mm^2^ s^−1^. As the CNF loading increased to 0.75, the λ increased to 0.26 Wm^−1^K^−1^, decreasing to 0.237 Wm^−1^K^−1^. This result demonstrates that the CNF loading up to 0.75 enhances λ of nanocomposites due to the well-ordered structure of the nanocomposite, resulting in good phonon-transporting efficiency of CNF in the VE-DDM resin [57]. After 0.75 wt% CNF, the reduction in λ is due to voids, cracks, and entanglements, as shown in SEM images (Fig. 4) [58]. The nanocomposites' thermal conductivity depends on the matrix's stiffness and morphology and the fibers' alignment [[59], [60], [61]]. Compared with the glass window (thermal conductivity = 0.96 Wm^−1^K^−1^) [62], the CNF-VE nanocomposites have a lower thermal conductivity, suggesting that CNF-VE nanocomposites have a potential for insulating material.

## 4 Conclusions

This study highlights the usability of lignin-derived vanillin epoxy (VE) resins with CNF to obtain environment-friendly and high-performance CNF-VE nanocomposites. The CNF-VE nanocomposites were prepared through the in-situ reaction of various CNF concentrations with VE resin and DDM hardener. 0.75CNF-VE nanocomposite exhibited higher tensile strength ∼127.78 ± 3.99 MPa and toughness ∼14.96 ± 0.71 MJ/m^3^ than the previously reported CNF-reinforcements epoxy nanocomposites. The CNF's OH groups accelerate the curing by forming the etherification and hydrogen bonds with the epoxy ring of resin, resulting in robust crosslinking and higher mechanical properties. CNF-VE nanocomposites showed almost similar onset thermal degradation stability to the VE-DDM thermoset, although *T*_*g*_ is gradually reduced with the increasing CNF wt%. Moreover, the water absorption of CNF-VE nanocomposites continuously increased (1.2–1.46 %) with the incorporation of CNF wt%, and the optimized 0.75CNF-VE nanocomposite exhibited 1.26 % water absorption and ∼91.79 ± 0.7° water contact angle. CNF-VE nanocomposites showed similar chemical resistance behavior with the VE-DDM thermoset in different organic solvents and the methanol/0.1 M HCl solution mixture at room temperature. Interestingly, the VE-DDM thermoset and CNF-VE nanocomposites were completely chemically degraded in the methanol/0.75 M HCl aqueous solution (8:2 ratios) at 60 °C temperature within 4 h. Moreover, CNF-VE nanocomposites demonstrated excellent UV-blocking ability in UVA, UVB, and UVC regions along with the haziness. The 0.75CNF-VE nanocomposite showed around four times lower thermal conductivity than glass. These high mechanical properties, hydrophobicity, UV shielding, low thermal conductivity, and environment-friendly characteristics of CNF-VE nanocomposites are attractive for building materials.

## Data availability statement

Data will be made available upon request.

## CRediT authorship contribution statement

**Bijender Kumar:** Writing – original draft, Methodology, Investigation, Conceptualization. **Samial Adil:** Formal analysis, Data curation. **Duc Hoa Pham:** Visualization, Validation. **Jaehwan Kim:** Writing – review & editing, Supervision, Project administration, Funding acquisition, Conceptualization.

## Declaration of competing interest

The authors declare that they have no known competing financial interests or personal relationships that could have appeared to influence the work reported in this paper.
