# Supplementary material for: Environment-friendly, high-performance cellulose nanofiber-vanillin epoxy nanocomposite with excellent mechanical, thermal insulation and UV shielding properties
Source: Heliyon. 2024 Jan 24;10(3):e25272. doi: 10.1016/j.heliyon.2024.e25272 (PMC10847658; doi:10.1016/j.heliyon.2024.e25272)
Supplement: Multimedia component 1 [file mmc1.docx]

**Supplementary Information**

**Environment-friendly, high-performance cellulose nanofiber-vanillin epoxy nanocomposite with excellent mechanical, thermal insulation and UV shielding properties**

Bijender Kumar, Samial Adil, Duc Hoa Pham, Jaehwan Kim*

Creative Research Center for Nanocellulose Future Composites, Department of Mechanical Engineering, Inha University, 100, Inha-ro, Michuhol-gu, Incheon 22212, South Korea

*Correspondence to: J. Kim (E-mail: jaehwan@inha.ac.kr)

**Preparation of Single Lap Joint Specimens**

A single lap joint (SLJ) test was performed to investigate the adhesion strength of the prepared VE-DDM resin. The adhesive area of the VE-DDM resins was 1 mm x 10 mm for SLJ specimens, and the thickness was around 2 μm. CNF film (thickness, 200 μm) was cut into a length of 40 mm and a width of 10 mm. Then, the CNF-VE specimens were cured in a vacuum oven under 150 °C for 1 hour, followed by 170 °C for 1 hour. A tensile test was performed to determine the adhesion strength of the SLJ specimens with a 1 mm/min pulling speed.


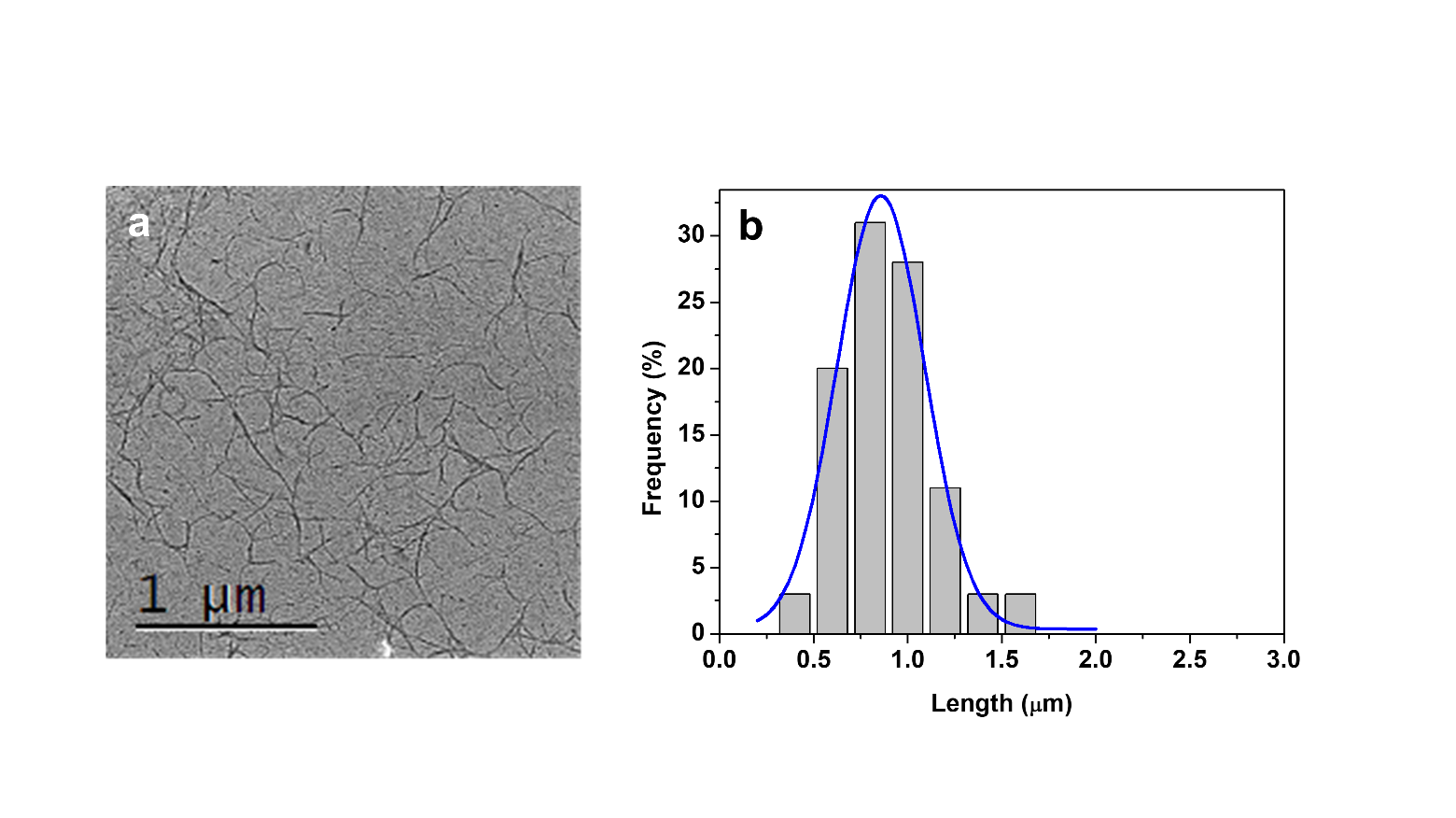


**Fig. S1**. (a) TEM image of CNFs and (b) CNF's size distribution.


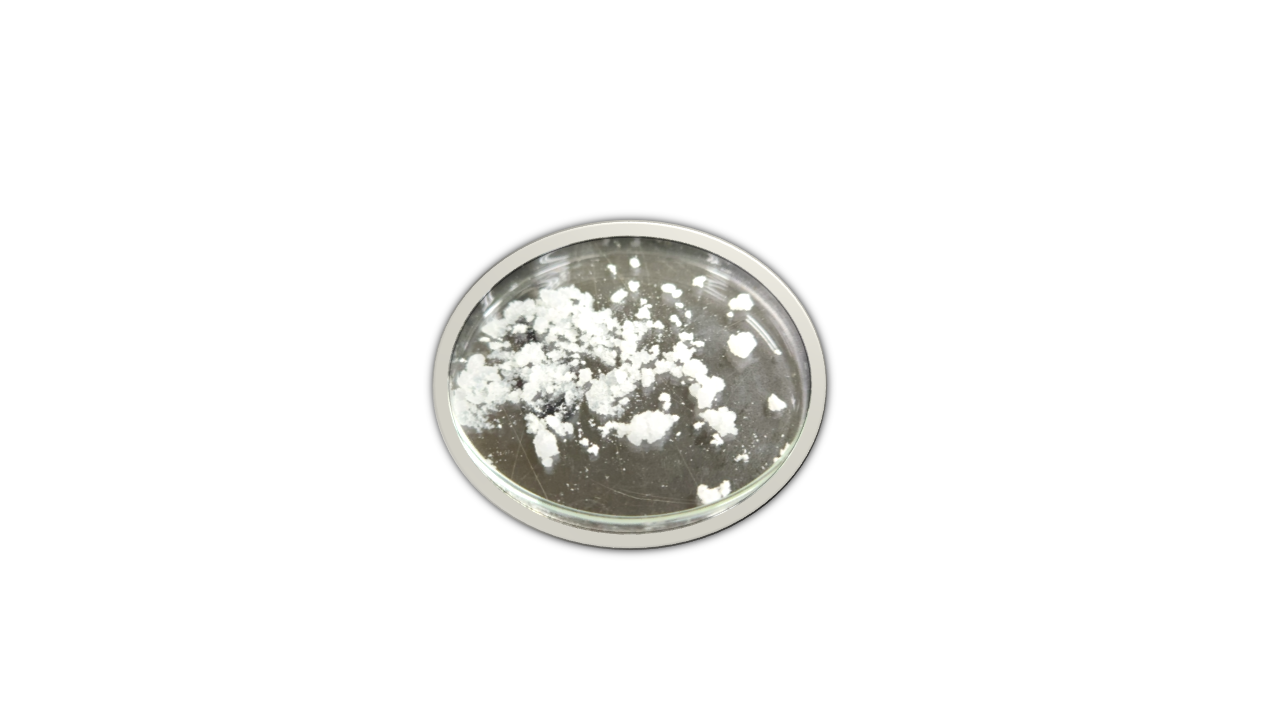


**Fig. S2**. The wet CNF image before compounding.


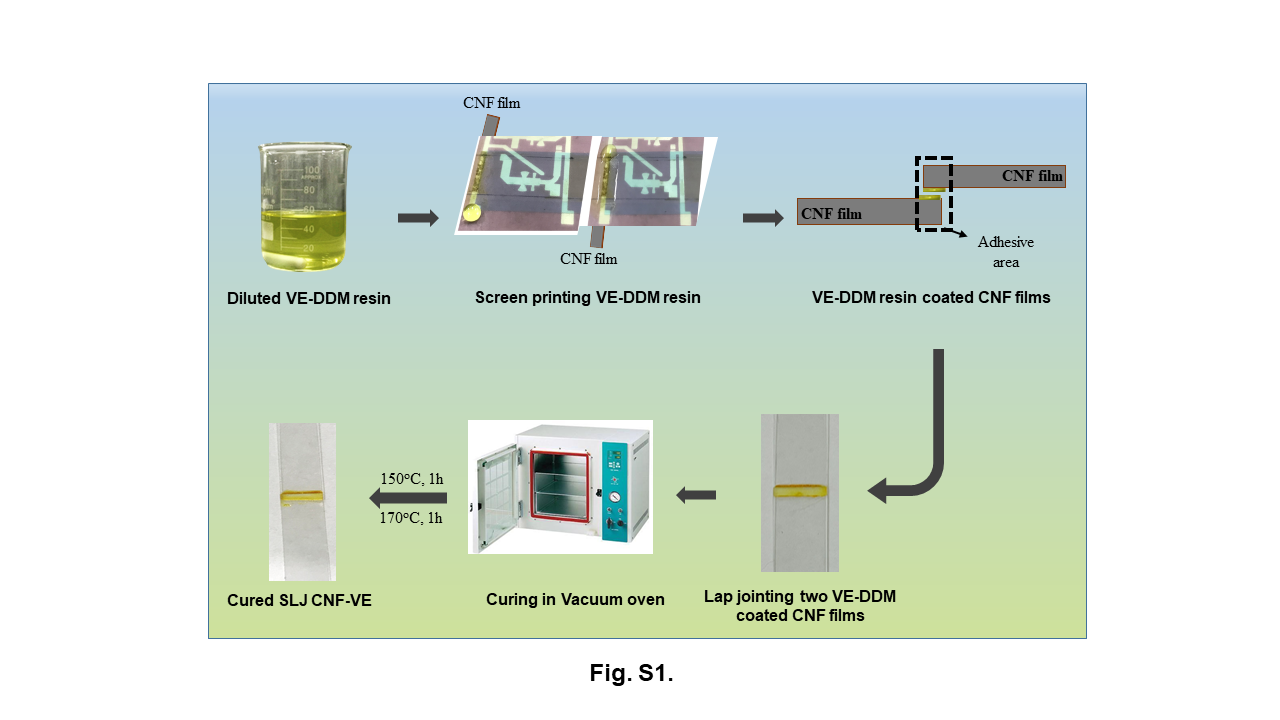


**Fig. S3.** Representative scheme for preparing SLJ samples of VE-DDM resin and CNF film.

**
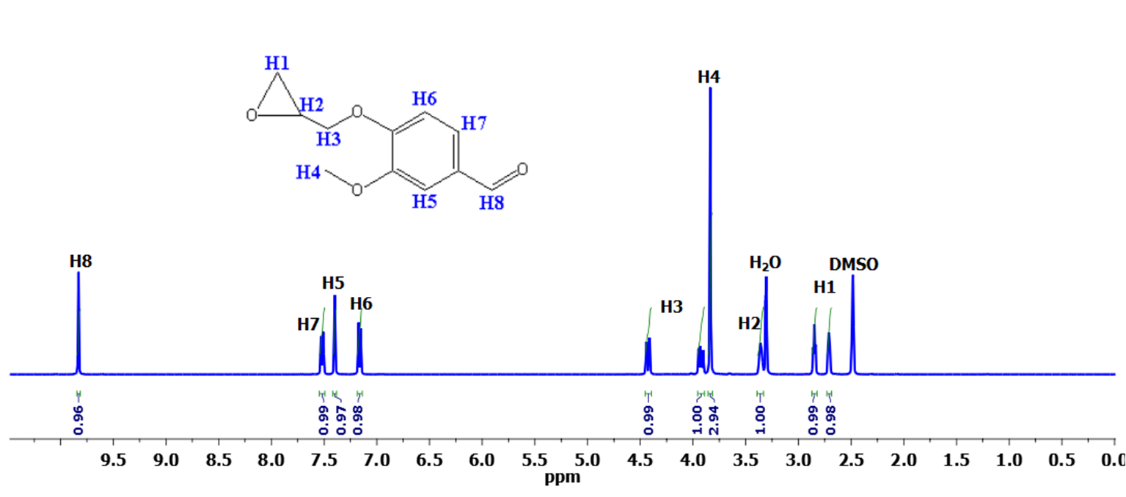
**

**Fig. S4.** ^1^H NMR spectra of VE.

**
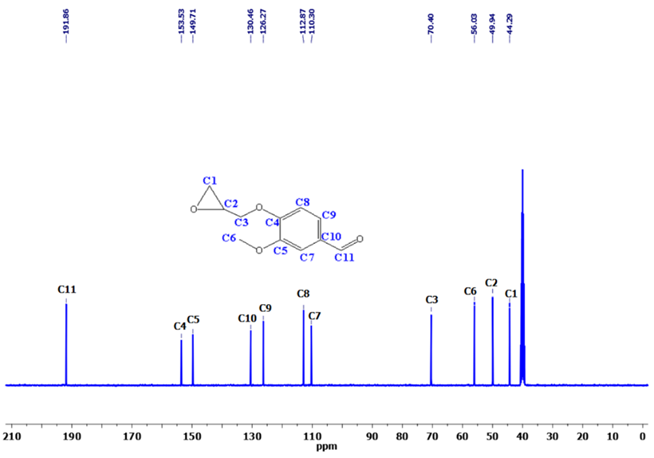
**

**Fig. S5.** ^13^C NMR spectra of VE.


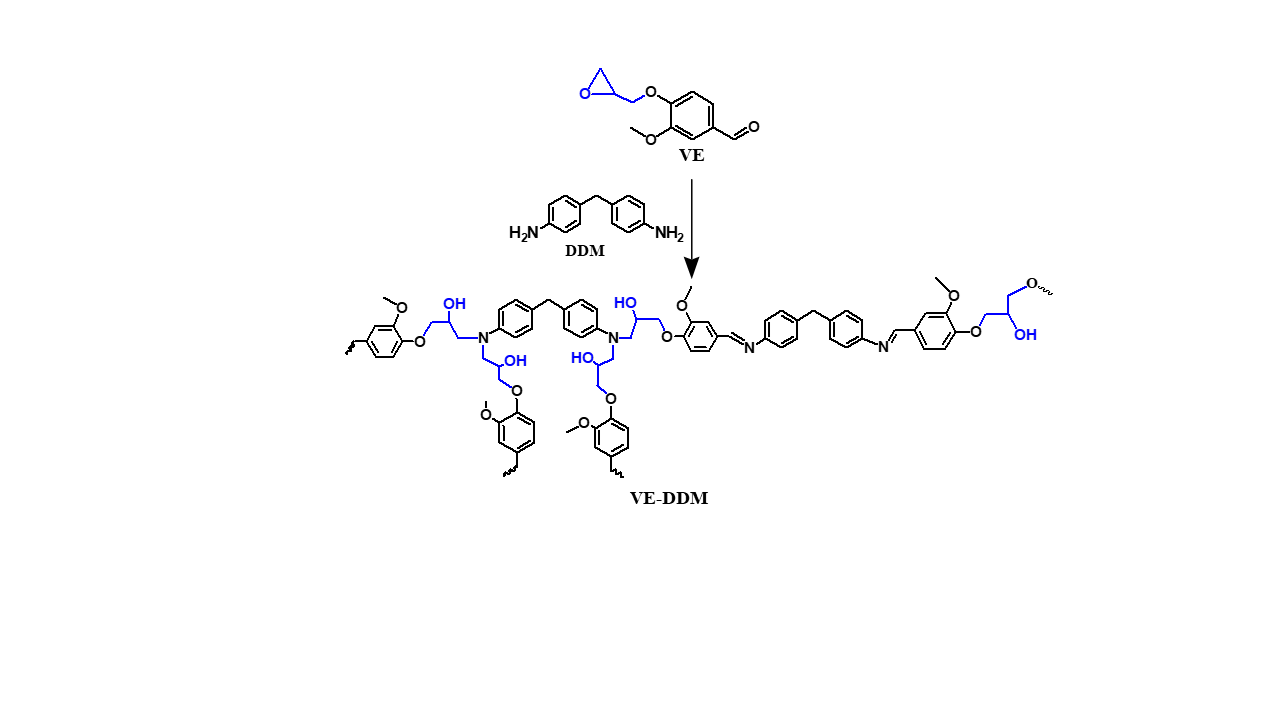


**Fig. S6.** The possible representative diagram of the curing of VE and DDM.


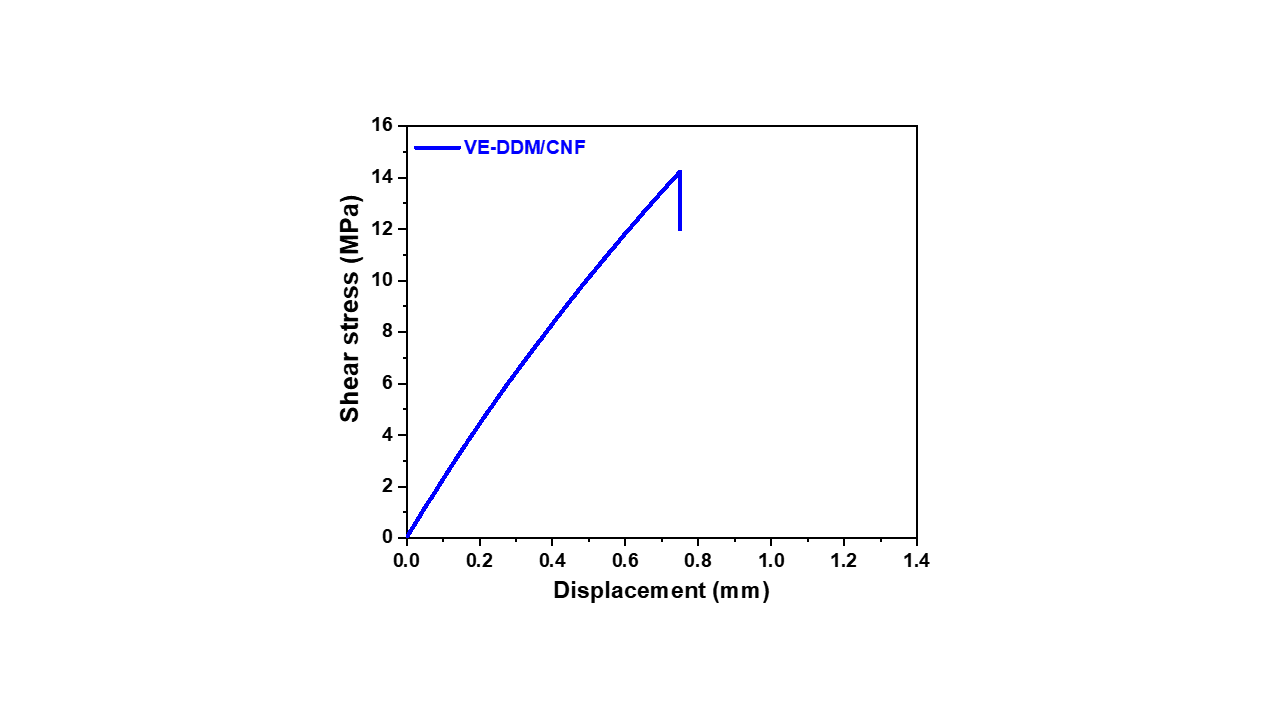


**Fig. S7.** Interfacial adhesion strength of VE-DDM resin with CNF films.

**
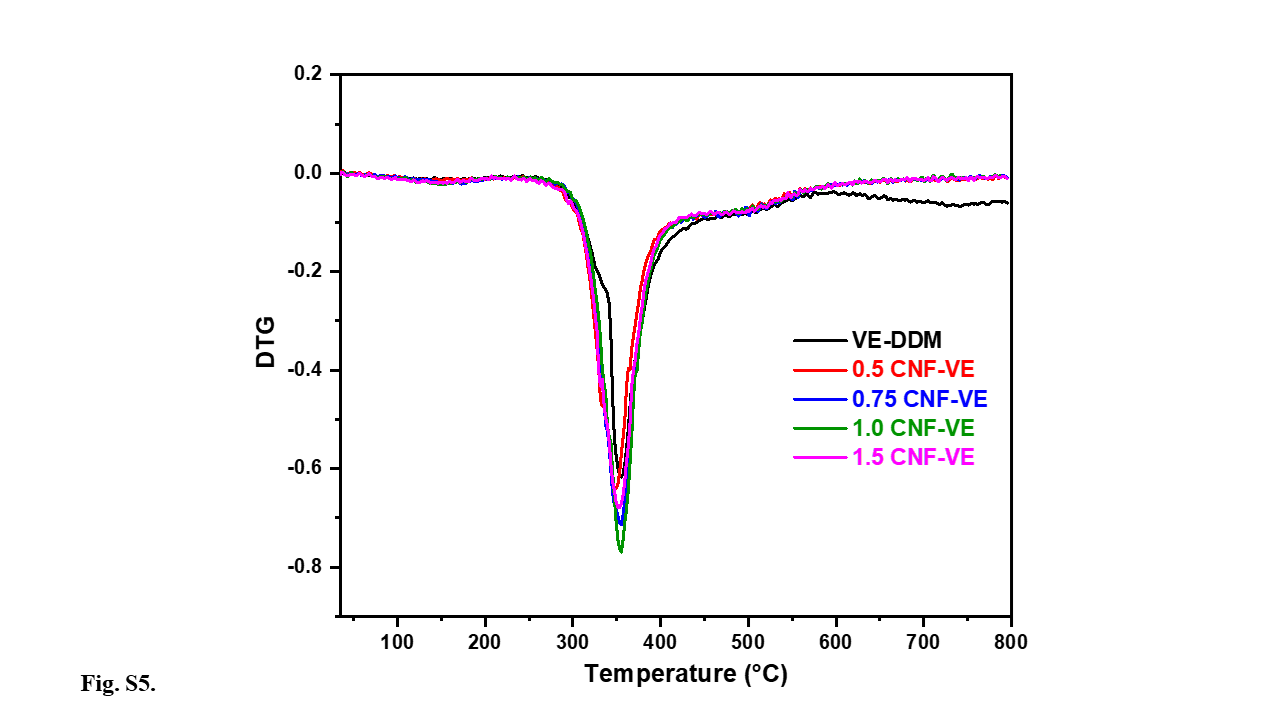
**

**Fig. S8.** DTG curves of VE-DDM thermoset and CNF-VE nanocomposites.

**
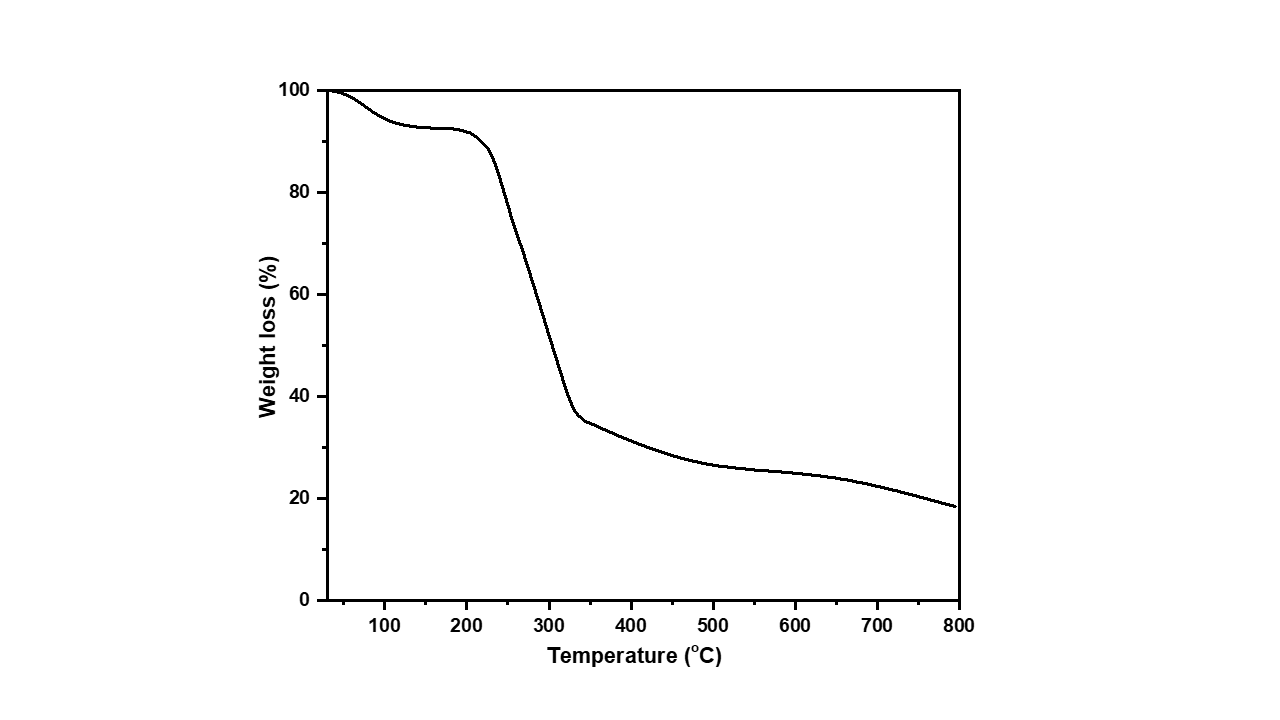
**

**Fig. S9.** TGA curve of CNF.

**
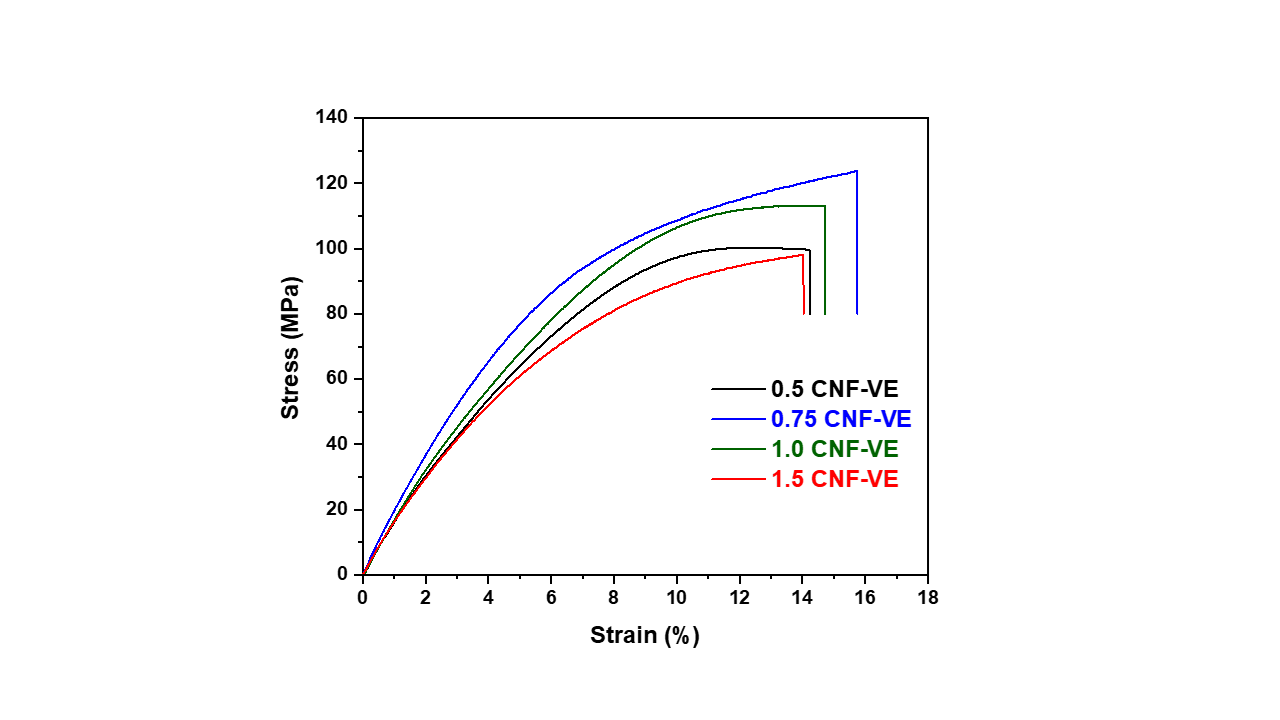
**

**Fig. 10.** Stress-strain curves of the CNF-VE nanocomposites after immersion in tap water for 7 days.

**Table S1.** Mechanical properties of wet CNF-VE nanocomposites after immersion in tap water for 7 days.

| Samples* | Stress (MPa) | Strain (%) | Tensile modulus  (GPa) | | Toughness (MJ/m^3^) |
| --- | --- | --- | --- | --- | --- |
| 0.5CNF-VE | 103.35±4.11^a,b)^ | 14.21±0.98^a)^ | 1.38±0.017^a)^ | | 10.09±0.92^a)^ |
| 0.75CNF-VE | 119.36±3.58^c)^ | 15.73±1.01^a)^ | | 2.09±0.23^b)^ | 13.61±0.89^b)^ |
| 1.0CNF-VE | 108.85±3.27^a,b)^ | 14.51±1.32^a)^ | 1.51±0.10^a)^ | | 10.14±0.62^a)^ |
| 1.5CNF-VE | 98.52±4.49^a)^ | 14.00±0.89^a)^ | 1.45±0.09^a)^ | | 9.37±0.43^a)^ |

*Same superscript letter data within the same column are not significantly (p > 0.05) different from Duncan's multiple range tests.
